# Trophic ecology of Mexican Pacific harbor seal colonies using carbon and nitrogen stable isotopes

**DOI:** 10.1371/journal.pone.0225889

**Published:** 2020-01-22

**Authors:** Maricela Juárez-Rodríguez, Gisela Heckel, Juan Carlos Herguera-García, Fernando R. Elorriaga-Verplancken, Sharon Z. Herzka, Yolanda Schramm

**Affiliations:** 1 Departamento de Biología de la Conservación, Centro de Investigación Científica y de Educación Superior de Ensenada, Ensenada, Baja California, México; 2 Departamento de Ecología Marina, Centro de Investigación Científica y de Educación Superior de Ensenada, Ensenada, Baja California, México; 3 Instituto Politécnico Nacional, Centro Interdisciplinario de Ciencias Marinas, Departamento de Pesquerías y Biología Marina, La Paz, Baja California Sur, México; 4 Departamento de Oceanografía Biológica, Centro de Investigación Científica y de Educación Superior de Ensenada, Ensenada, Baja California, México; 5 Facultad de Ciencias Marinas, Universidad Autónoma de Baja California, Ensenada, Baja California, México; University of Maryland Center for Environmental Science, UNITED STATES

## Abstract

There is limited information that provides a comprehensive understanding of the trophic ecology of Mexican Pacific harbor seal (*Phoca vitulina richardii*) colonies. While scat analysis has been used to determine the diet of some colonies, the integrative characterization of its feeding habits on broader temporal and spatial scales remains limited. We examined potential feeding grounds, trophic niche width, and overlap, and inferred the degree of dietary specialization using stable carbon and nitrogen isotope ratios (δ^13^C and δ^15^N) in this subspecies. We analyzed δ^13^C and δ^15^N on fur samples from pups collected at five sites along the western coast of the Baja California Peninsula, Mexico. Fur of natal coat of Pacific harbor seal pups begins to grow during the seventh month in utero until the last stage of gestation. Therefore pup fur is a good proxy for the mother’s feeding habits in winter (~December to March), based on the timing of gestation for the subspecies in this region. Our results indicated that the δ^13^C and δ^15^N values differed significantly among sampling sites, with the highest mean δ^15^N value occurring at the southernmost site, reflecting a well-characterized north to south latitudinal ^15^N-enrichment in the food web. The tendency identified in δ^13^C values, in which the northern colonies showed the most enriched values, suggests nearshore and benthic-demersal feeding habits. A low variance in δ^13^C and δ^15^N values for each colony (<1‰) and relatively small standard ellipse areas suggest a specialized foraging behavior in adult female Pacific harbor seals in Mexican waters.

## Introduction

The concept of trophic ecology is based on understanding the structure of feeding relationships among all trophic levels [1]. In addition, it is of great importance to ascertain the ecological role of top predators, such as the Pacific harbor seal (*Phoca vitulina richardii*), as they exert top-down control on lower trophic levels [2]. These interactions affect the structure, dynamics, and energy flux within a community or ecosystem [3, 4]. The harbor seal interacts both with members of the same subspecies and with other predators, which may result in competition for resources or niche partitioning, as it has been reported in other pinnipeds [5, 6].

The North Pacific harbor seal is distributed from Japan to Mexico [7], with its southernmost distribution recorded on nine islands and archipelagos, as well as on the west coast west of the Baja California Peninsula [8, 9]. Total counts based on photographs taken during aerial surveys conducted in 2009 yielded 4,862 individuals during the pupping season [9], representing only approximately 1.4% of the total abundance of the subspecies in the North Pacific, which is estimated at 375,100 individuals [10]. The annual life cycle of the harbor seal comprises foraging at sea and hauling out to breed, molt and rest, although the timing (start, peak, end, and duration) of these behaviors may vary depending on the latitude at which the colonies are located [11]. In Mexican colonies, seals start hauling out in mid-winter (late December-February) to give birth and breed [12], with pupping generally lasting from late December to the beginning of April, and longer in the southern than in the northern colonies of Mexico (*e*.*g*.14 weeks on San Roque Island and nine on Todos Santos Island, respectively) [12, 13]. Consequently, different degrees of synchronization occur among adult females from different colonies, whose lactation period lasts for three to four weeks [13]. Contrary to other seal species which fast during the breeding season (*e*.*g*. the northern elephant seal, *Mirounga angustirostris*) [14], the harbor seal tends to forage near its pupping and molting sites, no more than 45 km from shore [15].

Based on analysis of both scat and stomach contents, previous studies have suggested that the Pacific harbor seal is a generalist consumer with a high rate of consumption that depends on the seasonal and local abundance of benthic and pelagic prey (fish and cephalopods) [16–18]. While these traditional analyses enable prey identification up to a species-level taxonomic resolution, they are limited in that they correspond to a short time frame, that is, since the individual’s most recent foraging trips. Moreover, there is also the potential for misidentification or underestimation of prey due to the digestive process. In addition, prey items may be underestimated because the haul-out sites where scat is collected are only located in nearshore areas, so prey captured in offshore areas may not be in collected samples [19, 20]. Little is known regarding the trophic ecology of the Pacific harbor seal colonies along the Baja California Peninsula. Although there is a wide composition of prey species in the colonies studied here, the harbor seal tends to feed mostly on two to five items at each site, leading to the conclusion that this subspecies is a specialist consumer [21–24].

Due to the large temporal and spatial scales of their ecological niche, it is challenging to study the diet of marine mammals. Stable isotopes analyses (SIA) is a powerful tool for dietary reconstruction of predators [25, 26]. SIA provides information on assimilated prey over different time scales, depending on the tissue selected [27–29]. Tissues with a higher metabolic rate, *e*.*g*. blood, tend to have shorter integration times, while non-metabolic tissues such as fur, nails, and teeth retain the isotopic composition that reflects the food assimilated at the time of formation [26, 30–33]. The stable isotope ratio of carbon (δ^13^C) provides information on predator foraging areas and can be employed to determine primary producer sources in a trophic web. This is based on differences in isotopic fractionation due to taxon-specific photosynthetic pathways, variations in the isotopic composition of inorganic carbon caused by geochemical processes, and the differences between pelagic and benthic producers [31, 34]. Carbon isotope ratios of carbon sources at the base of the food web tend to display a gradient toward lighter values between coastal habitats and offshore regions, as well as in lower and higher latitudes, and when comparing pelagic and benthic food sources [34–36].

The stable isotope ratio of nitrogen (δ^15^N) is a proxy for trophic position, due to the predictable 2.5–3.4‰ ^15^N-enrichment occurring among each subsequent trophic level [36–38]. Tissue-to-diet isotope discrimination varies because of differences in metabolic routing of dietary components from tissue to tissue, animal physiology, and the nutritional quality of the individual’s diet [39, 40]. The δ^13^C and δ^15^N values observed in consumers are also influenced by the δ^13^C and δ^15^N values observed at the base of the food web.

The regional variation in stable carbon and nitrogen isotopes at the base of the food web occurs due to several factors, such as the predominant types of primary producers, nutrient and light levels, the intensity of the upwelling that results in the consequent transport of inorganic carbon and nitrogen, and the magnitude of fluvial, atmospheric or anthropogenic carbon and nitrogen inputs [34, 35, 41]. In the north-eastern Pacific Ocean, both δ^15^N and δ^13^C values at the base of the food web negatively correlate with latitude [30, 31, 41–43]. Moreover, the δ^15^N values of residual nitrate tend to be enriched in ^15^N near the tropics due to extensive denitrification regions in minimum oxygen zones (MOZs) [41, 44, 45]. Values of δ^13^C at the base of the food web are generally depleted in ^13^C at higher latitudes due to the lower sea surface temperature than that found at lower latitudes [35, 46], combined with light-limited low photosynthetic rates [24, 29].

Carbon and nitrogen stable isotope ratios have been used extensively to characterize feeding areas and movement patterns, and to track migration, residency, and food web structure for a variety of marine mammals. In the north-eastern Pacific Ocean, SIA has been successfully used to assess the foraging locations and preferences, trophic relationships and feeding grounds of several pinniped species, such as the harbor seal, the Steller sea lion (*Eumetopias jubatus*) [31], the northern fur seal (*Callorhinus ursinus*), the California sea lion (*Zalophus californianus*), and the northern elephant seal (*Mirounga angustirostris*) [26, 30, 42, 47].

Trophic niche width and foraging preferences have been characterized by the variance in the stable isotope ratios of populations of various taxa [48, 49]. The concept of ecological niche is defined as a hypervolume in n-dimensional space, in which an organism’s feeding behavior can be represented on environmental and bionomic axes [50]. Therefore, the isotopic niche provides a quantitative metric for representing the trophic niche [51–54].

Tissue selection for the analysis must take into account the period over which the isotopic composition of the tissue has been integrated [26]. The isotopic enrichment between marine mammal mothers and their pups is similar to that observed in a prey-predator relationship. Isotope analyses of tissue taken from pups have served as good proxies for maternal foraging habits [23, 30, 33], because fur grows in utero approximately from the seventh month onwards and continues to grow until the last stage of gestation [55]. The harbor seal sheds its lanugo in utero prior to birth [56], with its fur molting one year post-birth [57–59]; therefore, harbor seal pup fur reflects the feeding habits of its mother (*i*.*e*. an adult female with offspring) over a time frame equivalent to winter (December to March), the last months of gestation (~four months before birth). As pups are easier to capture and restrain than adults, they offer the opportunity to obtain a sufficiently large sample size to enable robust statistical testing.

The aim of the present study was to characterize the trophic ecology of adult *P*. *v*. *richardii* females by analyzing the stable isotopes of pup fur at five sites representative of its distribution in Mexican waters. We defined potential feeding grounds (benthic vs. pelagic; coastal vs. oceanic) during the pupping season, as well as the trophic niche width, position and overlap, and the degree of specialization in the studied colonies.

## Materials and methods

### Ethics statement

All Pacific harbor seal fur samples used in this study were collected and processed under special permits issued by the following: Dirección General de Vida Silvestre (General Wildlife Directorate), Secretaría de Medio Ambiente y Recursos Naturales (Ministry for the Environment and Natural Resources), Nos. SGPA/DGVS/12269/13 and SGPA/DGVS/08370/14; *Secretaría de Gobernación* (Ministry of the Interior), Nos. UG/211/0087/2014 and UG/211/01022/2014; and *Reserva de la Biosfera El Vizcaíno* (*El Vizcaíno* Biosphere Reserve) No. F00.DRPBCPN.-000027. All tissue samples were collected using non-invasive sampling techniques.

### Sample collection and processing

**Fig 1:**
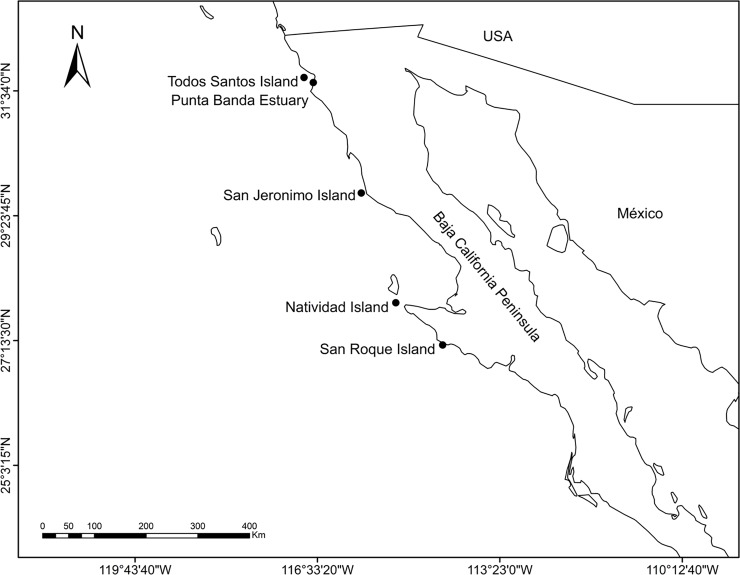
Location of sampling sites along the west coast of the Baja California Peninsula, Mexico, where fur sample were collected from Pacific harbor seal pups (n = 138) for stable isotope analysis.

Our sampling sites were located along the west coast of the Baja California Peninsula, in a north to south direction: Todos Santos (TSI), San Jerónimo (SJI), Natividad (NI); and, San Roque (SRI) islands. TSI and SRI are separated by 4° latitude (Fig 1). These islands form part of the Baja California Peninsula Pacific Islands Biosphere Reserve [60]. The Pacific harbor seal pups were sampled at these four islands and at the Punta Banda Estuary (PBE) (Fig 1).

From February to April 2015, we collected 138 fur samples from suckling harbor seal pups aged two to four weeks and of both sexes (TSI, n = 39; PBE, n = 12; SJI, n = 29; NI, n = 27; SRI, n = 31). Pup counts in 2009 for the islands were 56 for TSI, 99 for SJI, 124 for NI, and 181 for SRI [9], while 34 pups were estimated at PBE in 2011 [12]. We applied a minimally-invasive fur-collection method that involved shaving a 10x10 cm patch from the dorsal region of the pups using a stainless-steel razor, with the samples registering a ~3–5 g wet weight. Care was taken to avoid removing the fur follicle. Fur samples were washed with acetone and double-distilled water in the laboratory to remove salt and sand residue, and dried at 60ºC for 12 h to eliminate excess moisture. The fur samples were then homogenized in an agate mortar until a fine powder was obtained, which was then put in sealed tin capsules (5x9 mm) to achieve a target 0.5±0.05 mg dry weight.

### Sample analyses

The nitrogen and carbon isotope ratios in the fur were determined using a continuous flow mass spectrometer (Delta plus Advantage, Thermo Fisher Scientific, Bremen, Germany), coupled to an elemental combustion analyzer (model 4010 ECS, Costech Analytical Technologies, California, USA) at the Stable Isotope Laboratory, Centro de Investigación Científica y de Educación Superior de Ensenada (CICESE, or the Ensenada Center for Scientific Research and Higher Education, in Baja California, Mexico). The stable isotope values were expressed as δ (‰), in line with international standards, with Vienna Pee Dee Belemnite (VPDB) used for carbon, and atmospheric nitrogen (N_2_) used for nitrogen, in accordance with the equation:

$$\delta X=\left[\frac{R_{sample}}{R_{standard}}\right]∙1000$$ (1)

Where X is ^13^C or ^15^N and R_sample_ and R_standard_ are the molar quotients of the heavy isotopes over the light isotopes (^13^C/^12^C or ^15^N/^14^N) for the sample and laboratory standard, respectively. Secondary laboratory standards, including glutamic acid (δ^13^C: -26.39‰ and δ^15^N: -4.52‰), acetanilide (δ^13^C: -30.37‰ and δ^15^N: -0.83), seal pup lanugo (δ^13^C: -16.04‰ and δ^15^N: 15.32‰), and PF01 (δ^13^C: -15.94‰ and δ^15^N: 14.64‰) were routinely measured during sample analysis, with a high level of precision for all standards and both elements (<0.2 ‰ standard deviation). The laboratory protocol used is available at https://dx.doi.org/10.17504/protocols.io.2m3gc8n and in the Supplementary Information (S1 Protocol) for this study. The δ^13^C and δ^15^N values for each pup fur sample are also available in the Supplementary Information (S1 Dataset) for this study and at the Figshare repository, DOI: https://doi.org/10.6084/m9.figshare.8156273.

### Statistical analyses

The statistical tests were applied using the *Statistica* software (version 10.0, 2015), while the pups were grouped by sampling site. The δ^13^C and δ^15^N values were tested for normality using the Shapiro–Wilks test and for homoscedasticity, using the Levene test. Differences in δ^13^C values among the five sampling sites were assessed using one-way analysis of variance (ANOVA). Kruskal-Wallis analysis was used for δ^15^N values, because the nitrogen isotope ratios did not meet the assumptions of parametric statistics. When significant differences in δ^13^C were found among sampling sites, the Tukey’s test was applied, while, for δ^15^N, multiple comparisons of mean ranks were used as a non-parametric *a posteriori* test, in which a p-value < 0.05 was considered significant.

Based on isotopic variance, the dietary niche width is one of the metrics applied to measure the degree of specialization. To determine the isotopic niche width for the individuals from all the colonies studied here (four islands and one estuary), we first calculated the convex hull area (TA) of the δ^13^C-δ^15^N biplot for each colony [53]. The polygon was drawn around the outermost points of stable isotope values and is a direct measure of the area of isotopic niche space encompassed by a given colony. However, as the convex hull area can be sensitive to sample size [61], for comparative purposes, we also calculated isotopic ellipses using the Bayesian method SIBER (Stable Isotope Bayesian Ellipses in R) [53, 61], which was developed for the R package (R Development Core Team, 2008) and updated in February 2019 [62]. This method provides a robust estimate for comparing isotopic niche width, and is based on the calculation of standard ellipse areas (SEA) which are not as sensitive to limited sample sizes, as is the case for the convex hull model [61]. SIBER is frequently used in ecological studies with sample sizes n<50 [61]. Standard ellipse areas were corrected according to sample size (SEA_c_) and were calculated to nullify potential bias due to differences in sample size between colonies, with SEA_c_ the equivalent to the standard deviation in univariate cases, and less influenced by the extreme values of standard ellipses or convex hulls [61], containing approximately 40% of the data, SEA_c_ has a 95% credible interval, while the SEA_c_ differences among colonies were tested for statistical differences by comparing the SEA_c_ probability distributions for the colonies generated, as the outcome of 10^4^ resampling runs [61]. We calculated the overlap between two colonies by comparing their SEA_c_ in ‰ ^2^, in accordance with the following equation [61, 62]:

$$Overlap=\frac{overlap(A,B)}{total\;area\;A}$$ (2)

To test the above for geographical differences in isotopic values, we correlated the distance to the coast as well as the latitude and longitude (*i*.*e*., the east-to-west gradient) of each colony with the mean δ^13^C and δ^15^N values for each colony. The distance or proximity of each colony to the coast was calculated using the Haversine equation:

$$a={sin}^{2}\;\left(\frac{\Delta \varphi }{2}\right)+cos\varphi_{1}∙cos\varphi_{2}∙{sin}^{2}\;\left(\frac{\Delta \lambda }{2}\right)$$ (3)

$$c=2∙atan2\;(\sqrt{a},\sqrt{\left(1-a\right)}$$

d=R∙c

Where:

φ = Latitude

λ = Longitude

R = Radius of the Earth, assumed to be 6,371 km

d = Distance between two points

### Relationship with diet

Stable isotopic values reflect the composition of the assimilated diet [63], but not necessarily the ingested diet [22]. Therefore, information relating to the ingested diet is not necessarily recorded, while isotope niche widths can lead to the underestimation of the dietary niche. Conversely, species richness may not reflect the actual consumption of individual prey.

The trophic spectrum of the harbor seal’s diet was correlated based on species richness estimates derived from the identification of the hard parts of fish and cephalopods found in scat [22], which were cross-referenced with our estimated isotopic niche width (*i*.*e*., number of prey species for each colony *vs*. the SEA_c_ and the TA). Species richness data recorded during the pupping season were considered for TSI, SJI, NI, and SRI [22], while the data for PBE were excluded because scat was collected during the molting season, and the seals’ diet tends to vary from season-to-season or, even, month-to-month [21]. The prey species identified from hard parts were also plotted in terms of their trophic level, Index of Prey Importance (IIMPi) [22] and their habitat type (demersal, bathydemersal, benthic, benthopelagic, or pelagic) [64].

## Results

### Inter-individual variation

**Fig 2:**
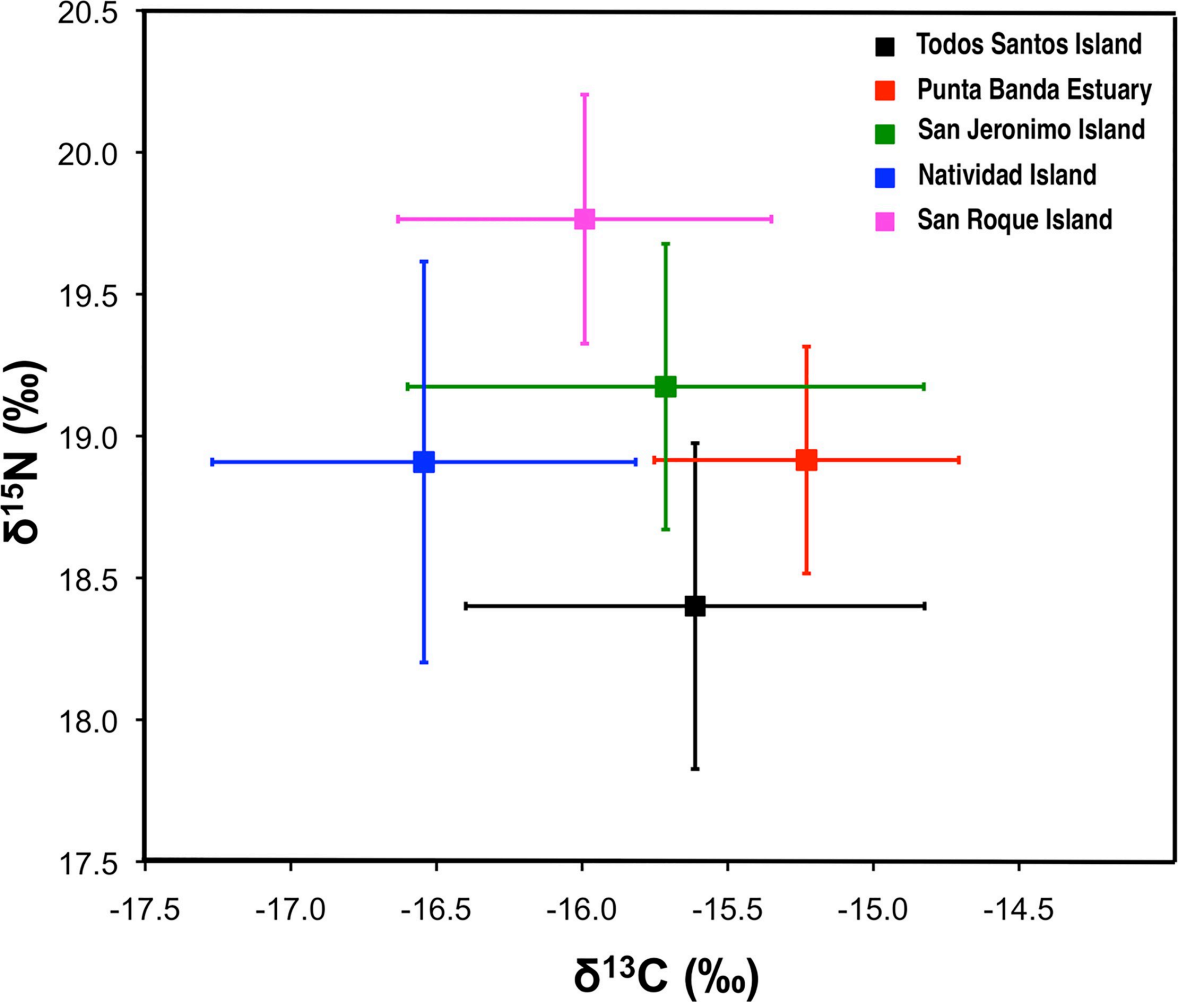
δ^15^N and δ^13^C values (mean ± SD) of Pacific harbor seal pup samples at different sites along the west coast of the Baja California Peninsula (TSI = Todos Santos Island, PBE = Punta Banda Estuary, SJI = San Jerónimo Island, NI = Natividad Island, and SRI = San Roque Island).

The δ^15^N values from individual Pacific harbor seal fur samples ranged between 16.8 and 21.5‰ (mean ± SD: 19.0 ± 0.7‰; Table 1, Fig 2). The SRI sampling site recorded the highest mean δ^15^N value, while TSI recorded the lowest. The δ^13^C values ranged between -18.3 and -13.6‰ (mean ± SD: -15.9 ± 0.8‰). The broadest range of δ^13^C values was exhibited by the pups sampled at TSI (-17.1 to -13.6‰), while the broadest δ^15^N values were found at the SRI colony (19.0 to 21.6‰). Individuals sampled at PBE showed the most limited range of isotopic values for both elements. Those individuals sampled at NI showed the most depleted carbon isotope ratios, in contrast to PBE, where the most enriched carbon isotope values were found.

**Table 1:** δ^13^C and δ^15^N (mean ± SD) values for the pup fur of Pacific harbor seals sampled at five sites, given with their geographical location and distances to shore along the western coast of the Baja California Peninsula, Mexico.

	Distance		δ^13^C	δ^15^N
Sampling sites	to shore	n	mean± SD	mean ± SD
	(km)		(‰)	(‰)
**Todos Santos Island**	16.46	39	-15.6± 0.8	18.40±0.6
(31°47’ 59'' N, 116°47’ 20'' W)				
**Punta Banda Estuary**	0.29	12	-15.23±0.5	18.92±0.4
(31° 42’ 47'' N, 116° 40’ W)				
**San Jerónimo Island**	9.44	29	-15.71±0.9	19.17±0.5
**(**29°47' 33'' N, 115°47'29'' W)				
**Natividad Island**	9.80	27	-16.54±0.7	18.91±0.7
**(**27°52' 55'' N, 115°11'33'')				
**San Roque Island**	1.8	31	-15.99±0.6	19.76±0.4
**(**27°8' 48 '' N, 114°22'40'' W)				

Overall, significant differences were found in the δ^13^C values for the pup fur samples collected at five colonies along the western coast of the Baja California peninsula (ANOVA, F_4, 133_ = 9.321, p<0.001; Fig 2). For of the δ^13^C values, NI was statistically different from all other sites, while SRI was statistically different from PBE and NI (Tukey´s test, p <0.05). There were also significant differences in δ^15^N values among the colonies (KW, H_4, 138_ = 68,795, p <0.001; Fig 2), while statistical differences were found between TSI in comparison with SJI, NI, and SRI. SRI was significantly different from all other study sites (non-parametric *a posteriori* test, Table 2).

**Table 2:** Results of the paired comparisons of the nitrogen isotope ratios of Pacific harbor seal pup fur among five sampling sites along the west coast of the Baja California Peninsula (TSI = Todos Santos Island, PBE = Punta Banda Estuary, SJI = San Jerónimo Island, NI = Natividad Island, and SRI = San Roque Island).

	*A posteriori test*			
	p-value			
	TSI	PBE	SJI	NI
**PBE**	0.54			
**SJI**	< .01	1.00		
**NI**	0.03	1.00	1.00	
**SRI**	<0.01	< .01	< .01	< .01

### Trophic niche

**Fig 3:**
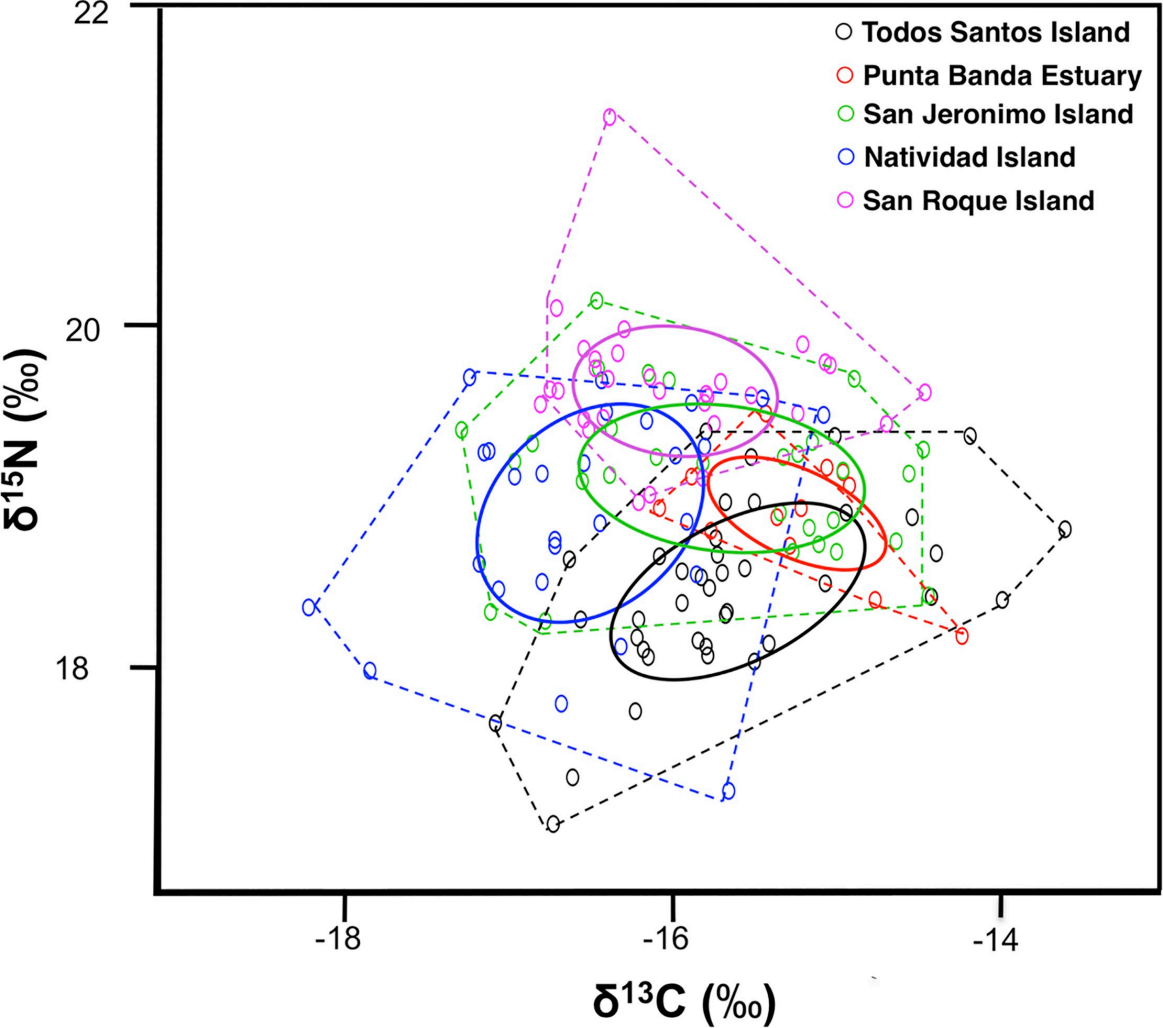
Individual δ^15^N and δ^13^C values of pup fur samples for pup fur samples from Pacific harbor seals at five sampling sites along the west coast of the Baja California Peninsula. Solid lines represent the standard ellipses corrected for sample size (SEA_c_) and dashed lines represent the convex hull areas.

Variance in carbon and nitrogen stable isotope values within sampling sites was low (<1‰), both for δ^13^C (TSI: ±0.6, PBE: ±0.3, SJI: ±0.8, NI: ±0.5, and SRI: ±0.4 in ‰) and for δ^15^N (TSI: ±0.3, PBE: ±0.3, SJI: ±0.3, NI: ±0.5, and SRI: ±0.3‰). The convex hull total areas (TA) were largest for TSI and NI (5.1 and 5.9, respectively), while PBE presented the smallest TA (1) (Fig 3). The highest overlap in isotopic space was between SJI and NI (3.0), followed by NI and TSI (2.7); while the smallest overlap found was between SRI and PBE (0.1) (Table 3).

**Table 3:** Areas and overlap of convex hulls calculated for the carbon and nitrogen isotope ratios for Pacific harbor seal pup fur sampled at five sites along the west coast of the Baja California Peninsula (TSI = Todos Santos Island, PBE = Punta Banda Estuary, SJI = San Jerónimo Island, NI = Natividad Island, and SRI = San Roque Island).

	Convex hull areas (‰^2^)	Overlap (‰^2^)			
		TSI	PBE	SJI	NI
**TSI**	5.14				
**PBE**	1.00	0.98			
**SJI**	4.70	2.24	0.95		
**NI**	5.86	2.66	0.51	3.00	
**SRI**	3.21	0.23	0.14	1.67	0.87

**Fig 4:**
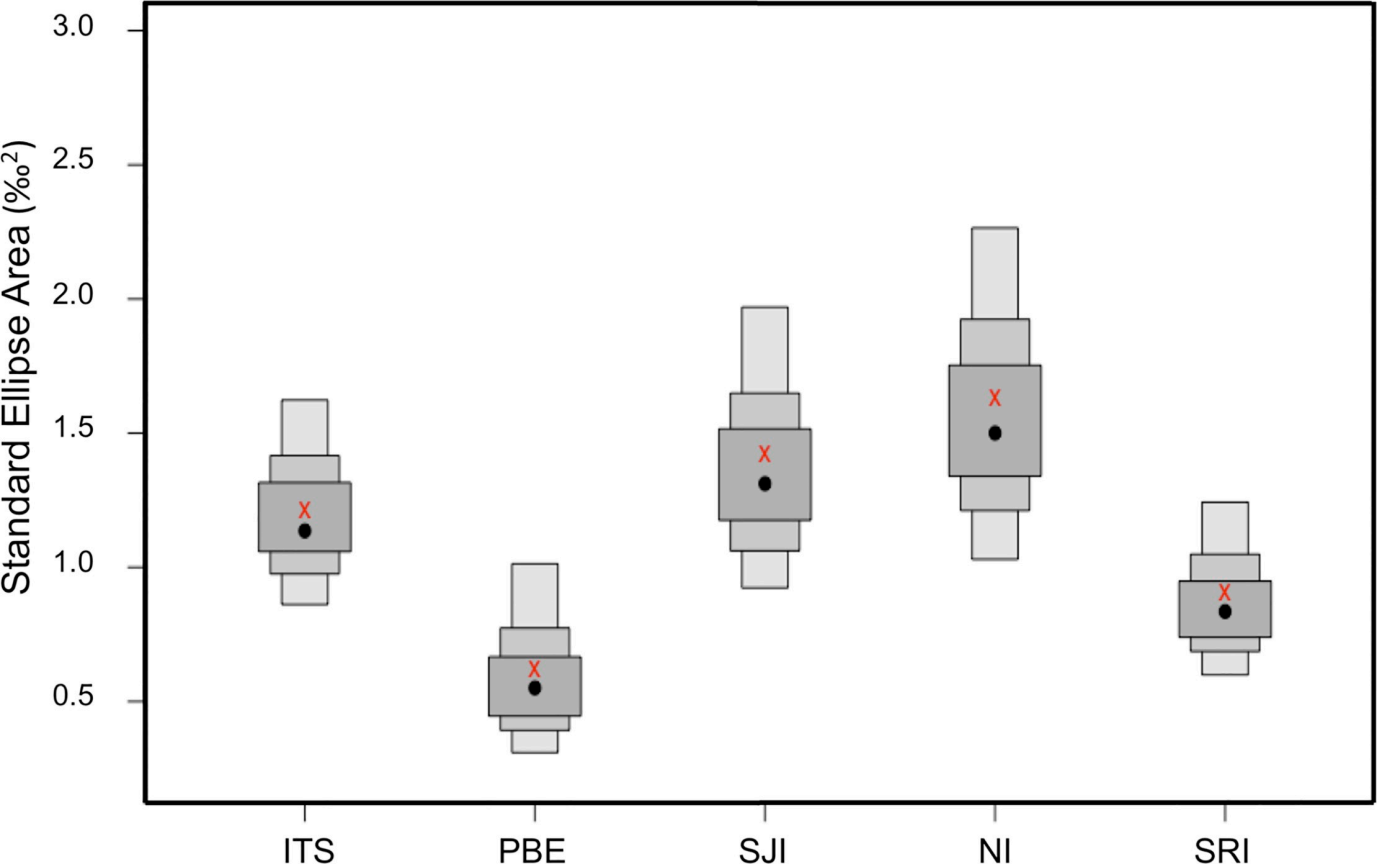
SIBER density plot, with credibility intervals (50% dark grey boxes, 75% mid grey boxes, 100% light grey boxes), for the Bayesian-generated ellipses (SEA—black dots) for the Pacific harbor seal pup isotope data overlaid with the sample size corrected for uncertainty in the estimates (SEA_c_—red crosses). The distribution in the estimates of the standard ellipse area (‰) is based on 10^6^ resampling runs.

Based on the SEA_c_ values, NI presented the largest trophic width (1.6) and PBE the smallest (0.6) (Fig 4, Table 4). A large overlap (0.5) was found between SJI and NI, followed by PBE and SJI (0.5), and TSI and PBE (0.3). The smallest trophic overlap found for this metric was between TSI and SRI.

**Table 4:** Areas and overlap of Standard Ellipse Areas corrected for the sample size (SEA_c_) and calculated for the carbon and nitrogen isotope ratios of Pacific harbor seal pup fur at five sampling sites along the west coast of the Baja California Peninsula (TSI = Todos Santos Island, PBE = Punta Banda Estuary, SJI = San Jerónimo Island, NI = Natividad Island, and SRI = San Roque Island).

	SEA_c_ (‰^2^)	Overlap (‰^2^)			
		TSI	PBE	SJI	NI
**TSI**	1.22				
**PBE**	0.62	0.29			
**SJI**	1.42	0.21	0.45		
**NI**	1.64	0.05	0	0.53	
**SRI**	0.91	<0.01	<0.01	0.29	0.12

### Geographical variation

No correlation was found between the proximity to shore and the δ^15^N and δ^13^C pup fur values. However, we observed a high explained variance (r = 0.58, p = 0.081) for carbon isotope ratios, a positive and significant correlation between mean δ^15^N values for both colonies and longitude (r = 0.78, p = 0.046), and an enrichment trend in ^15^N towards the south, but which was not significant (r = 0.57, p < 0.087). In contrast, the correlations between mean δ^13^C values and proximity to the shore, as well as latitude and longitude were not significant.

### Relationship with diet

**Fig 5:**
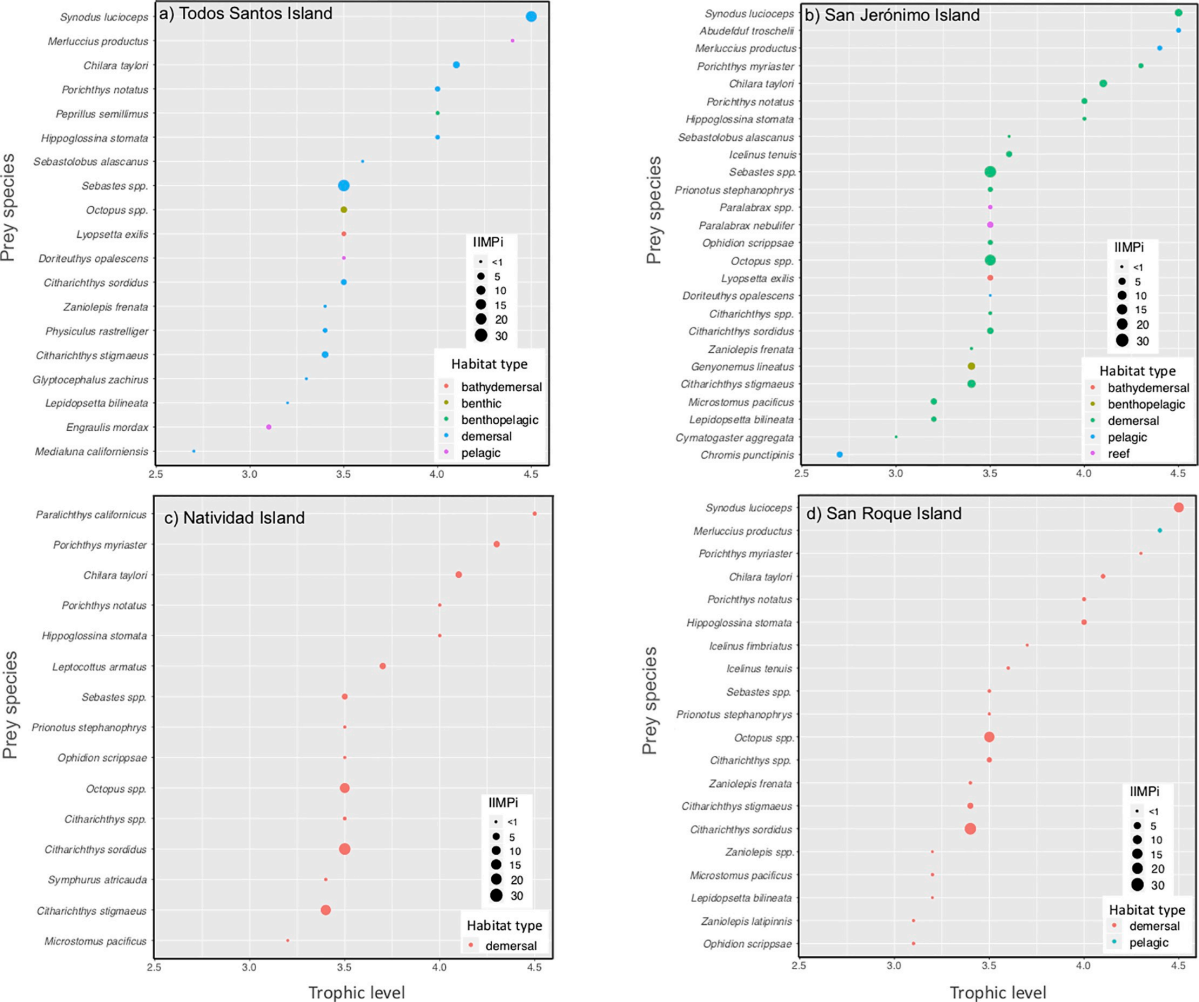
Harbor seal prey species identified by hard remain analysis of scat [22], with their corresponding estimated trophic level (x axis), Importance Index (IIMPi, which varies according to circle sizes), and predominant habitat type (colored circles).

There were no significant correlations between SEA_c_ and TA estimates in our study with species richness identified by hard remains previously [22] (SEA_c_: r = -0.40, p = 0.75; TA: r = -0.80, p = 0.33; Table 5). This absence of correlation is due to the fact that San Jerónimo and San Roque islands had the highest species richness (26 and 20 taxa, respectively, Fig 5B and 5D), which did not coincide with the largest TAs and SEA_c_ (Table 5). In addition, we did not find a significant correlation between the number of prey species shared between sites and their corresponding overlap areas (r = 0.21, p = 0.689, Fig 5, Table 6).

**Table 5:** Species richness (number of prey species) in four colonies with their corresponding Standard Ellipse Area (SEA_c_) and Total Area (TA) calculated from pup fur isotope analysis.

Site	SEA_c_ (‰^2^)	TA (‰^2^)	Species richness
**Todos Santos Island**	1.22	5.14	19
**San Jerónimo Island**	1.42	4.70	26
**Natividad Island**	1.64	5.86	15
**San Roque Island**	0.91	3.21	20

**Table 6:** Number of prey species shared between sites, with their corresponding overlap areas (‰^2^).

SITES	Number of shared prey species	Overlap areas (‰^2^)
ITS-ISJ	14	0.21
ITS-IN	6	0.05
ITS-ISR	11	0
ISJ-IN	10	0.53
ISJ-ISR	12	0.29
IN-ISR	11	0.12

The absence of significant correlation between species richness and overlap areas between colonies reflects different prey preferences among the colonies (IIMPi). For example, demersal species, such as *Sebastes* spp., *Synodus lucioceps* and *Octopus* spp., appear with larger IIMPi in TSI, SJI and SRI than NI (Fig 5).

## Discussion

The factors controlling isotopic incorporation into tissue have to be taken into account by trophic ecology studies inferred from stable isotopes. The present study used fur, a metabolically inert tissue that reflects the isotopic composition of the prey assimilated during biosynthesis [65, 66]. Dietary changes are recorded in fur as a function of the time required for isotope equilibration between the diet assimilated and formation of the fur [67–69]. As explained in the introduction of the present paper, harbor seal pup fur is a good proxy for the mother's feeding habits in a time-frame equivalent to winter (December to March), based on the last months of gestation in the region of study [12].

To date, only a few studies have analyzed stable isotope values in fur samples taken from harbor seal pups pertaining to Mexican colonies [23, 70, 71], with one of those studies [72] reporting higher mean δ^13^C values for the NI colony (δ^13^C: -15.4±0.6‰) than those obtained by the present study (δ^13^C: -18.3±0.7‰). This discrepancy could be caused by the fact that the sampling for the studies was conducted in different sampling years (April 2013 vs. December 2014-February 2015), meaning that, therefore, there was either a distinct difference in isotopic composition at the base of the food web or distinct difference in prey availability. A warm water anomaly in the northeast Pacific, which started off the coast of Alaska and subsequently spread to southern Baja California [73], occurred from winter 2013–2014 to summer 2015 and caused a 1-4°C increase in sea surface temperature. This event may have changed the composition and structure of the region’s trophic webs by modifying the abundance and distribution of the organisms and also, therefore, the availability of harbor seal prey. The study discussed above [72] collected samples in April 2013, approximately ten months before the warm water anomaly began, while our samples were collected in January-February 2015, when the anomaly was already well established.

Isotopic niche width can be considered a measurement of a species’ degree of specialization [74]. The low variance in carbon and nitrogen stable isotopes in pup fur (<1‰) suggests that female harbor seals adhere to specialized foraging habits [48, 75]. In fact, previous studies indicate that female harbor seals prefer a narrow range of prey species, with three to five dominant species documented in their diet [48]. The results of the present study suggest that the largest convex hull total area calculated for NI indicates more generalized foraging habits or a lower degree of specialization, compared to the other colonies studied here. In contrast, PBE presented the smallest convex hull area, low variance, and smaller ellipse areas, and, therefore, the highest degree of specialization. This finding may be due to the high IIMPi of three fish species found in the diet of the colony’s members—the California lizardfish (*Synodus lucioceps*), Verrill's two-spotted octopus (*Octopus bimaculatus*) and the California market squid (*Dorytheutis opalescens*) [21]. However, up to 23 species of ichthyofauna have also been documented as prey for the individuals of this colony [76]. The PBE colony occupies a small geographical area [12], with a previous study finding that the prey species of its Pacific harbor seal population were significantly different from those of the TSI, which is located less than 20 km away from PBE [21], probably because the PBE harbor seal colony seems to feed only on species found in the estuary [77]. In addition, the central region of the Baja California Peninsula shows a lower number of fish habitats and species groups compared with the northern region [78], which may explain the smaller trophic niche of the PBE colony. This smaller isotopic niche means that the PBE colony is of ecological relevance, given that specialist species are more sensitive to changes in resource availability than generalists [77, 79, 80].

The differences in δ^13^C values among Mexican Pacific harbor seals colonies seem to reflect different site-specific oceanographic conditions that have caused variations in the isotopic baseline, although a latitudinal pattern in the δ^13^C values of harbor seal pup fur was not evident. The greatest discrepancy was found between NI and PBE. With PBE located inshore and NI near Punta Eugenia, the latter colony is more strongly influenced by the California Current and the North Equatorial Countercurrent than the former. This oceanic influence is consistent with the lower δ^13^C values found at NI [72], which contrast with the higher δ^13^C values found at PBE, that is a coastal lagoon, and result from the higher contribution of benthic carbon sources or benthic macrophytes, which are significantly enriched in ^13^C in relation to phytoplankton [46, 81]. The δ^13^C values were highest in the coastal colonies at TSI and PBE, which probably reflects the significant presence of benthic-demersal prey items [82].

Isotopic differences in δ^15^N values among all colonies were significant, with the exception of TSI and PBE, which are located in close proximity to each other. The higher δ^15^N values found at SRI likely reflect a latitudinal ^15^N enrichment of the baseline towards the south, caused by regional denitrification processes in the tropical equatorial Pacific and the subsurface transport of enriched nitrate to the north by the California Counter Current [83]. The high δ^15^N pup fur values from SRI, the southernmost site in the present study, likely reflects the greater contribution of heavy nitrate. The 1‰ increase in the mean isotope ratios of the fur taken at SRI and TSI, colonies separated by four degrees of latitude, is consistent with the equatorward ^15^N enrichment shown in other studies [8, 39, 84].

### Isotopic comparison among pinniped species

Comparing our isotopic data with fur samples taken from other pinniped species in the northeastern Pacific may be useful for understanding the trophic ecology of this subspecies at the southern end of its distribution [23, 31, 71, 85, 86]. The δ^15^N values observed in the present study (19.0±0.7) were low compared to those previously reported for California sea lions (*Zalophus californianus*) in the Gulf of California (21.8 ±0.7‰) [39, 87], although they were higher than those reported for Galapagos sea lions (*Zalophus wollebaeki*, 12.94±0.43‰, Table 7).

**Table 7:** Stable isotope values (mean ± SD) in fur taken from different pinniped species in the eastern Pacific Ocean and the Gulf of California, Mexico.

Common name	Species	Age	Sampling year	Locations	δ^13^C	δ^15^N	Reference
California sea lion	*Zalophus californianus*	Pups	2008	Gulf of California	-15.9±0.5	21.8±0.7	[39]
Galápagos sea lion	*Zalophus wollebaeki*			Galápagos Islands	-14.5±0.5	13.1±0.5	[39]
Pacific harbor seal	*Phoca vitulina richardii*	Pups (2mo)	2012	Natividad Island	-15.40±0.6	19.10±0.3	[72]
Galápagos sea lion	*Zalophus wollebaeki*	Pups (2mo)	2009	Galápagos Islands (avg.)	-16.2±0.3	12.94±0.4	[88]
				Cabo Douglas	-15.2±0.5	13.12±0.3	[88]
				Caamaño	-16.42±0.2	12.92±0.6	[88]
				Post Office	-16.59±0.3	12.79±0.4	[88]
				Malecon	-16.62±0.3	12.96±0.5	[88]
Pacific harbor seal	*Phoca vitulina richardii*	Pups (1–2 mo)	2013	Natividad Island	-15.4±0.6	19.1±0.3	[70]
Northern elephant seal	*Mirounga angustirostris*	Pups (1–2 mo)		San Benito Islands	-17.2±0.8	17.6±1.3	[70]
Guadalupe fur seal	*Arctocephalus philippii townsendi*	Pups (1mo)	July 2013	Guadalupe Island	-17.6± 0.3	18.4±0.4	[89]
				San Benito Islands	-17.1±0.4	18.8±0.4	[89]

The δ^13^C values for the fur taken from Guadalupe fur seals at Guadalupe Island were approximately 2.6‰ lighter (-18.4±0.4, Table 4) than the values obtained for Mexican Pacific harbor seals. The δ^15^N (17.6±1.3) and δ^13^C (-17.2±0.8) values for the fur taken from Northern elephant seals (*Mirounga angustirostris*) at San Benito Islands were lower and more variable than our measurements for harbor seals (Table 4) [70].

The comparatively lower δ^15^N values found by the present study on the west coast of the Baja California Peninsula revealed differences among trophic web baselines at other sites, such as the Gulf of California and the Galapagos islands [39, 87].

The differences in δ^13^C values found among the fur samples taken from Guadalupe fur seals at Guadalupe Island and the values obtained from Mexican Pacific harbor seals most likely occur because Guadalupe Island is located at an oceanic location 250 km from the coast of the peninsula and the fact that Guadalupe fur seals forage on offshore squid [89]. The difference between the δ^15^N values obtained for Northern elephant seals and those found in the samples taken in the present study may indicate a latitudinal gradient, as the elephant seal forages in northern waters (as far north as the Gulf of Alaska) and offshore areas on the west coast of North America [14, 75]. The differences observed in δ^13^C values may result from the effect of both latitude and distinct diets, in that the Northern elephant seal feeds primarily on squid [14]. However, this possibility has to be considered with caution, as we did not analyze isotopic values at the food web baseline. In addition, unlike the elephant seal, the harbor seal does not undertake long-distance migrations and long feeding trips, instead foraging in nearshore habits, which most likely explains the relatively higher δ^13^C values in the former than the latter. *P*.*v*. *richardii* shows a high degree of site fidelity [90, 91], which is especially the case for females during the breeding season, when they forage no more than 30 km from their haul-out site [91–93]. All the evidence discussed here suggests that the Pacific harbor seal follows local foraging habits in Mexican waters, which contrasts with other pinnipeds in the region, which forage in open waters further offshore.

### Relationship with diet

This study did not find a significant correlation between the trophic spectrum and isotopic niche width (SEA_c_), which may be due to the fact that hard parts remaining in scat reflect the diet ingested over one or even several days prior to sampling, while the SEA_c_ reflects the diet assimilated over the preceding months, during which period tissue synthesis occurred. The harbor seal seems to prefer three to five prey species within a wide potential trophic spectrum, with its prey’s different habitats and trophic levels possibly increasing isotopic variability. For example, previous hard remain analysis [22] shows that the harbor seal prey species with the highest IIMPi for TSI were the Redfish (*Sebastes* spp.) and the California lizardfish (*Synodus lucioceps*), which adhere to demersal habits. Fur samples taken from the TSI colony yielded relatively high δ^13^C values, which is consistent with feeding on prey that are at least partially dependent on benthic carbon sources [39, 70].

### Limitations

The more precise interpretation of the isotopic values within or among animal tissue requires information yielded by three sources of isotopic variation: 1) the isotopic composition of potential prey; 2) verification as to how isotopic fractionation occurs between sources and animal tissue; and, 3) the feeding period as reflected by specific tissue [26].

1) The present study attempted to correlate SIA results obtained with prey identified from hard remains [22], although no significant correlation was found. Future studies would be more successful if they were able to characterize the isotopic baseline and the isotopic composition of potential prey. A thorough characterization of an isotopic baseline requires extensive sampling that adequately captures spatial and temporal sources of variation or nutrient sources, as well as primary producers, and the prey. Although the intensity of the sampling effort needed and the remoteness of our sampling sites made it prohibitive to adequately characterize the baseline, specific compounds have recently been analyzed to obtain the baseline isotopic values in food webs. For example, nitrogen and carbon compound-specific isotopic analyses (CSIA) of some aminoacids have been shown to reflect the isotopic baseline [94].

2) In phocid seals, very different isotope fractionation has been reported among tissue types, including blood serum, red cells, and fur, ranging from 0.8 to 3.1 ‰ [68]. Little is known about isotope fractionation in the mother-to-foetus nutritional transference that occurs during gestation. While in many pinniped species, a pup’s isotopic composition has been reported as higher than its mother’s [39, 87, 95], this has not been reported either for this subspecies or in this tissue (fur) sampled by the present study. Measuring mother-to-pup fractionation requires the handling of adult females and their pups in the wild, which has proven to be difficult.

3) In this study, we assumed that the harbor seal pup fur has an isotopic composition that reflects the last few months of gestation. Furthermore, we assumed that pup fur begins to be isotopically enriched while being biosynthesized *in-utero*, which begins during the seventh month of gestation. Therefore, the pups’ isotopic values will correspond to the maternal foraging habits during the last three months of gestation, rather than the diet ingested over the entire 11–12 month gestation period.

As our results solely explain the trophic ecology of those adult females that were mothers at the time of sampling, no information was obtained on other females, males, and other age categories (juveniles or adults). Energy requirements may limit foraging behavior in other pinniped species, mostly during the breeding season [96].

## Conclusions

The significant differences in both δ^15^N and δ^13^C revealed by this research among Mexican Pacific harbor seal colonies, coupled with previously established coastal-oceanic gradients for δ^13^C values and a regional latitudinal gradient for δ^15^N values, suggest specific local biogeochemical conditions that lead to the differences in isotopic baseline observed in the pups. Based on δ^13^C values that are relatively high, we can conclude that the feeding habitat of adult female harbor seals is mainly benthic-demersal and nearshore in nature, with slight differences occurring due to type of prey consumed in each colony. The relatively higher δ^15^N values recorded at the southernmost island (SRI) and the midpoint of SJI imply a latitudinal ^15^N-enrichment at the base of the food web, which was, apparently, mostly influenced by denitrification and microbial processes. Thus, the variability observed in the δ^15^N values obtained in the present study may involve taxonomic similarity among prey throughout the colonies, over a time-scale isotopically reflected in the fur. An alternative explanation may be that there is an isotopic similarity among different prey, because of their demersal habitat, which causes an underestimation of the degree of resource partitioning among colonies. The low isotopic variance observed in our results indicated that the Pacific harbor seal is a specialist consumer.

## Supporting information

S1 Protocol Laboratory protocol. Laboratory protocol for δ^15^N and δ^13^N stable isotope analysis in harbor seal pup fur samples.
(PDF)

S1 Dataset Stable carbon and nitrogen values. δ^13^C and δ^15^N values for each pup fur sample to analyze trophic ecology of Pacific harbor seal females at five sites west off the Baja California Peninsula, Mexico.
(XLSX)
