# Supplementary material for: Trophic ecology of Mexican Pacific harbor seal colonies using carbon and nitrogen stable isotopes
Source: PLoS One. 2020 Jan 22;15(1):e0225889. doi: 10.1371/journal.pone.0225889 (PMC6975529; doi:10.1371/journal.pone.0225889)
Supplement: S1 Protocol — Laboratory protocol for δ15N and δ13N stable isotope analysis in harbor seal pup fur samples. (PDF) [file pone.0225889.s001.pdf]

# CICESE’s laboratory protocol for carbon and nitrogen stable isotope analysis in harbor seal pup fur

In the Stable Isotope Laboratory at CICESE (Ensenada Center for Scientific Research and Graduate Education, Mexico) animal tissues (pup fur from Pacific harbor seals, *Phoca vitulina richardii*) were ground for isotopic determinations. Samples were stored in a dissecator until they were loaded into a Costech® Carrousel. Samples were combusted at high temperature (1000˚C) in a pure oxygen atmosphere in an elemental analyzer coupled to a Finnigan MAT Delta V Advantage continuous flow stable isotope mass spectrometer.

Stable carbon and nitrogen ratios are expressed as $\delta^{13}C$ or $\delta^{15}N$ according to the following equation:

$\delta^{13}C$ or $\delta^{15}N$ (‰) = [Rsample/Rstandard)–1]* 1000 (1)

where R is $^{13}C/\ ^{12}C$ or $^{15}N/\ ^{14}N$.

Isotopic values of carbon and nitrogen are reported relative to Pee Dee Belemnite and atmospheric nitrogen standards, respectively. The accuracy of isotopic measurements was verified using secondary standard reference materials (Glutamic acid, a pure carbonate Merck and the laboratory internal references Lanugo for N, and CH94 for C).

Protocol available at https://dx.doi.org/10.17504/protocols.io.2m3gc8n
